# Alexithymia, not autism, is associated with impaired interoception

**DOI:** 10.1016/j.cortex.2016.03.021

**Published:** 2016-08

**Authors:** Punit Shah, Richard Hall, Caroline Catmur, Geoffrey Bird

**Affiliations:** a MRC Social, Genetic and Developmental Psychiatry Centre, Institute of Psychiatry, Psychology and Neuroscience, King's College London, University of London, UK; b Department of Psychological Sciences, Birkbeck College, London, UK; c Department of Psychology, Institute of Psychiatry, Psychology and Neuroscience, King's College London, University of London, UK; d Institute of Cognitive Neuroscience, University College London, UK

**Keywords:** Autism, Alexithymia, Interoception, Interoceptive awareness, Body awareness

## Abstract

It has been proposed that Autism Spectrum Disorder (ASD) is associated with difficulties perceiving the internal state of one's body (i.e., impaired interoception), causing the socio-emotional deficits which are a diagnostic feature of the condition. However, research indicates that alexithymia – characterized by difficulties in recognizing emotions from internal bodily sensations – is also linked to atypical interoception. Elevated rates of alexithymia in the autistic population have been shown to underpin several socio-emotional impairments thought to be symptomatic of ASD, raising the possibility that interoceptive difficulties in ASD are also due to co-occurring alexithymia. Following this line of inquiry, the present study examined the relative impact of alexithymia and autism on interoceptive accuracy (IA). Across two experiments, it was found that alexithymia, not autism, was associated with atypical interoception. Results indicate that interoceptive impairments should not be considered a feature of ASD, but instead due to co-occurring alexithymia.

## 1 Introduction

Interoception, the perception of the internal state of one's body, has been relatively neglected by clinical psychology and neuroscience. However, there is renewed interest in interoception, driven by biologically-grounded predictive coding models of interoception (Seth, 2013) and realization of the clinical relevance of atypical interoception (Bird & Cook, 2013). Atypical interoception has been associated with various illnesses, such as obesity, anxiety and depression (Barrett & Simmons, 2015), but perhaps the most well-developed interoceptive theory is that Autism Spectrum Disorder (ASD), particularly the social symptoms characteristic of the disorder, is a result of (oxytocin-mediated) interoceptive dysfunction (Quattrocki & Friston, 2014).

The limited empirical evidence as to the interoceptive ability of those with ASD provides equivocal evidence for the interoception account of ASD however, with studies demonstrating both impaired and enhanced interoceptive ability in this population. Fiene and Brownlow (2015) found that autistic^1^ adults report significantly lower body and thirst awareness relative to neurotypical controls, leading them to conclude that “adults with ASD reported a unified *hypo*reactivity to elements of the interoceptive sense” (Fiene & Brownlow, 2015, p. 715). Garfinkel et al. (2016) also report that adults with ASD show impaired interoception, and speculate that this leads to impaired emotional sensitivity and anxiety in ASD. In contrast, however, using an objective measure of interoception in which participants are asked to count their heartbeats (Schandry, 1981), children with ASD showed enhanced rather than diminished interoception relative to a neurotypical control group (Schauder, Mash, Bryant, & Cascio, 2015).

While both hyper- and hypo-sensitivity to interoceptive signals may be consistent with Quattrocki and Friston's (2014) interoception account, there are other factors which may have led to the inconsistent results concerning interoceptive ability in ASD. For example, the superior heartbeat counting performance demonstrated by the autistic children in Schauder et al.'s (2015) study was only demonstrated at the longest test durations, raising the possibility that their enhanced performance was due to non-specific factors, such as the increased attention to counting tasks or enhanced time perception demonstrated by children with autism (e.g., Wallace & Happé, 2008). Of most relevance to the present study, however, is the fact that previous studies do not account for the elevated levels of alexithymia in the autistic population.

Alexithymia is characterized by difficulties in recognizing emotions from internal bodily sensations. Its prevalence in the general population is relatively low (10%; Salminen, Saarijärvi, Äärelä, Toikka, & Kauhanen, 1999), yet alexithymia frequently co-occurs in individuals with ASD (50%; Hill, Berthoz, & Frith, 2004). Elevated levels of alexithymia are also reported in a variety of psychiatric (Bird & Cook, 2013) and neurological (Ricciardi, Demartini, Fotopoulou, & Edwards, 2015) conditions. Importantly, however, alexithymia is a completely independent construct, with its own etiological and neurocognitive basis (Fitzgerald & Molyneux, 2004). Although the precise neurocognitive mechanisms underlying alexithymia remain under investigation, there is good evidence to suggest – given the reliance of interoception on emotion (Clark, 2013, Seth, 2013, Wiens, 2005) – that alexithymia is associated with impaired interoception (Herbert, Herbert, & Pollatos, 2011). Interestingly, it may be the case that alexithymia is associated with increased attention to bodily signals, at least according to self-report data (Ernst et al., 2014), making the reduced interoceptive accuracy (IA) in the condition even more striking.

In addition, there is a growing body of research showing that co-occurring alexithymia underpins several socio-emotional atypicalities (e.g., empathic deficits, emotion recognition difficulties) once regarded as features of ASD (Bird et al., 2011, Bird et al., 2010, Cook et al., 2013, Foulkes et al., 2015, Silani et al., 2008). In line with this evidence, Brewer, Happé, Cook, and Bird (2015) propose that, where observed in ASD, signs of atypical interoception are due to co-occurring alexithymia. Under this account, the interoceptive impairments previously observed in groups with ASD would be due to the fact that the ASD group contained more alexithymic individuals than the control group.

As such, it remains unclear whether atypical interoception is associated with ASD or alexithymia, or whether each condition has an independent impact on interoceptive ability. In addition, this question needs to be addressed while controlling for other clinical traits previously reported to be associated with IA (see Farb et al., 2015), and by ensuring that any relationship between ASD and interoception is not due to non-specific factors such as attention or time estimation (TE) ability. In Experiment 1 we therefore sought to examine the relative impact of alexithymia and autism on IA, while controlling for depression, anxiety, body mass index (BMI), sustained attention and TE ability.

## 2 Experiment 1

### 2.1 Method

#### 2.1.1 Participants

Thirty-eight adults (20 female) without a psychiatric diagnosis aged between 19 and 75 years (*M*_age_ = 29.1 years, SD = 12.7) participated in the study. Participants had normal or corrected-to-normal vision, and provided informed consent. Ethical clearance was granted by the local committee. Participants were recruited from a local database to ensure an adequate range of scores on trait measures of autism and alexithymia (see Section 2.1.2.4).

#### 2.1.2 Measures & procedure

##### 2.1.2.1 Interoception

The Heartbeat Tracking task (Schandry, 1981) was used to measure interoception. Participants were seated upright in a quiet room and were required to close their eyes and silently count their heartbeats during four intervals of varying duration (25, 35, 45, and 100 sec). The order of intervals was randomized across participants. Heartbeat signals were acquired using a finger pulse oximeter (Contec Systems CMS-50D+; Qinhuangdao, China) attached to the index finger, while the other arm was at rest. Participants were instructed not to measure their pulse by any means other than “concentrating on their heart beats”.

##### 2.1.2.2 Time Estimation (TE)

It has been suggested that performance on the Heartbeat Tracking task may be influenced by one's ability to estimate an elapsed time interval, and neural correlates of interoception and time duration overlap within insular cortex (Wittmann, 2013). While in the position described in Section 2.1.2.1, participants were therefore instructed to judge the duration of three randomized intervals (19, 37 and 49 sec, e.g., Ainley, Brass, & Tsakiris, 2014).

##### 2.1.2.3 Anthropometrics

Participants' height and weight were measured to calculate BMI.

##### 2.1.2.4 Questionnaire measures

Participants completed the 20-item Toronto Alexithymia Scale (TAS-20; Bagby, Parker, & Taylor, 1994), Autism-spectrum Quotient (AQ; Baron-Cohen, Wheelwright, Skinner, Martin, & Clubley, 2001), Beck Depression Inventory (BDI-II; Beck, Steer, & Brown, 1996), and the Spielberger State/Trait Anxiety Inventory (STAI; Spielberger, Gorsuch, Lushene, Vagg, & Jacobs, 1983). State anxiety was measured immediately before the Heartbeat Tracking task.

#### 2.1.3 Scoring and data analysis

IA was taken as performance on the Heartbeat Tracking task (Garfinkel, Seth, Barrett, Suzuki, & Critchley, 2015). Performance was quantified on a scale between 0 and 100% using the following transformation: $1/4\sum (1-\left(|{\text{recorded}\;\text{number}\;\text{of}\;\text{heartbeats}}_{\text{interval}}-{\text{counted}\;\text{number}\;\text{of}\;\text{heartbeats}}_{\text{interval}}|/{\text{recorded}\;\text{heartbeats}}_{\text{interval}}\right))\times 100$. Higher scores are indicative of better IA. TE score was also computed on a scale of 0–100% using this formula: 1/3∑(1−(|actualelapsedtime−estimatedelapsedtime|/actualelapsedtime))×100. Higher scores are indicative of better TE ability.

### 2.2 Results and discussion

All measures were found not to differ significantly from a normal distribution (Kolmogorov–Smirnov tests, all *p*s > .24) and there were no significant outliers. IA ranged between 12.70 and 98.80% (*M* = 69.12%, SD = 19.78%), consistent with previous studies that have used this task. Performance on the TE task followed a similar distribution (*M* = 73.13%, SD = 22.81%), indicating that it was matched in difficulty to the Heartbeat Tracking task and therefore an appropriate control measure.

Correlational analyses showed that alexithymia (TAS-20 score) was associated with IA (*r* = −.36, *p* = .025). In contrast, there was no correlation between IA and autistic traits (AQ score; *r* = −.09, *p* = .59), TE score, depression, BMI, state or trait anxiety measures (see Supplemental materials; Table S1). TAS-20 scores were, in line with previous research (e.g., Cook et al., 2013), correlated with AQ (*r* = .56, *p* < .001). Participant age was correlated with autistic traits (*r* = .57, *p* < .001) and alexithymia (*r* = .35, *p* = .034). Data were therefore entered into a hierarchical regression analysis to test the relative contribution of trait autism and alexithymia to IA, while controlling for age, sex, depression, anxiety, BMI, TE, and alexithymia and autism, respectively.

Participant age (years), sex (1 = male; 2 = female), depression score, state and trait anxiety scores, TE score and BMI were entered into the first step of the regression model, alexithymia scores in the second step, and autism scores in the third. Trait anxiety scores were predictive of IA at a trend level (*β* = .50, *t* = 1.92, *p* = .066), but no other variables in the first step reached significance (other *p*s > .10). When alexithymia was added (step 2), it was found to be predictive of IA (*β* = −.47, *t* = −2.64), and significantly increased the variance in IA accounted for by 16.4%, *F*(1,27) = 6.95, *p* = .014. Conversely, the addition of autistic trait scores (step 3) did not improve the model, leading to a non-significant increase of 0.2%, *F*(1,26) = .07, *p* = .80, while alexithymia remained the only significant predictor of IA, *β* = −.45, *t* = −2.20, *p* = .037. We ran a further hierarchical regression, entering autistic traits in step 2 and alexithymia in step 3. Here, autistic traits failed to significantly improve the model, accounting for an additional 4.7% of the variance in IA. Adding alexithymia led to a significant change in *R*^2^, increasing the explained variance by 11.8%, *F*(1,26) = 4.86, *p* = .037.

These results suggest that alexithymia, and not autism, is associated with impaired IA. Autistic traits failed to explain a significant proportion of variance in IA. In contrast, and as predicted by the ‘alexithymia hypothesis’ (Brewer et al., 2015), trait alexithymia was associated with IA, even after accounting for autistic traits and other variables that have previously been associated with IA.

However, it could be argued that autistic traits in the general population are not a valid proxy to estimate the performance of individuals with a clinical diagnosis of ASD (Ruzich et al., 2015) validated by gold-standard research diagnostic tools (e.g., Autism Diagnostic Observation Schedule – ADOS; Lord et al., 2000). Experiment 2 addressed this potential limitation.

## 3 Experiment 2

### 3.1 Introduction and method

In Experiment 2, we recruited nineteen adults diagnosed with ASD by an independent clinician and a control group of age-, gender-, and IQ-matched neurotypical individuals (see Supplemental materials). Autism severity was quantified in the autistic group using the ADOS, on which all participants met criteria for ASD. Autistic traits were found to be significantly higher in the ASD than in the control group. Each group contained individuals with (TAS-20 score of ≥61), and without, alexithymia, such that the two groups were closely matched on this dimension (Supplemental materials; Table S2). It was predicted that there would be no group difference in IA – because the groups were matched for alexithymia – but that alexithymia would predict IA. Methods were as used in Experiment 1.

### 3.2 Results and discussion

**Fig. 1:**
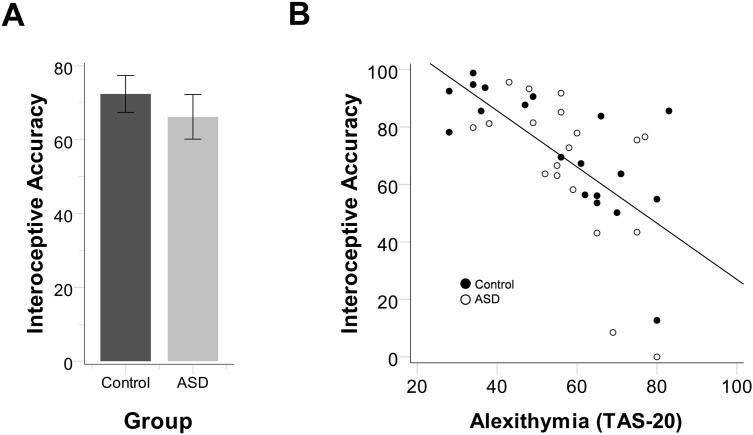
(A) Interoceptive accuracy compared between groups with and without Autism Spectrum Disorder (ASD) matched for alexithymia. (B) The simple correlation between the 20-item Toronto Alexithymia Scale (TAS-20; Bagby et al., 1994) and interoceptive accuracy (*r* = −.64, *p* < .001).

All measures were found not to differ significantly from a normal distribution (Kolmogorov–Smirnov tests, all *p*s > .16). There was no significant difference between the ASD (*M* = 81.96%, SD = 14.16%) and control (*M* = 74.91%, SD = 19.78%) groups on TE, *t*(36) = 1.26, *p* = .21, *d* = .41, consistent with previous evidence for intact time perception in ASD (e.g., Wallace & Happé, 2008). Most importantly, there was no group difference on IA, *t*(36) = .79, *p* = .43, *d* = .26 (Fig. 1A). Within the ASD group, the correlations between ADOS score and IA (*r* = −.22, *p* = .38) or TE (*r* = .18, *p* = .45) were not significant. Similarly, there was no relationship between autistic traits and IA in either group (ASD: *r* = −.05, *p* = .85; control: *r* = −.27, *p* = .27). See Supplemental materials (Table S3) for other correlations.

Results are consistent with Experiment 1 and support the prediction that, if groups are matched for alexithymia, ASD is not associated with impaired interoception. Following Experiment 1, alexithymia was highly correlated with IA, *r* = −.64, *p* < .001 (Fig. 1B), with little correlation between the AQ and IA (*r* = −.20, *p* = .23). TAS-20 scores were again correlated with AQ (*r* = .37, *p* = .024), although participant age, in this sample, was not correlated with autistic traits (*r* = .26, *p* = .12) or alexithymia (*r* = .14, *p* = .41).

We performed the same regression analysis described in Experiment 1 with the addition of IQ in the first step of the hierarchical regression. No variables in the first step reached significance (all *p*s > .12). When alexithymia was added to the model it was found to be predictive of IA (*β* = −.64, *t* = −4.13), significantly increasing the variance accounted for by 28.8%, *F*(1,27) = 17.03, *p* < .001. The addition of autistic traits failed to improve the model, leading to a non-significant increase of 1.4%, *F*(1,26) = .83, *p* = .37, while alexithymia remained the only significant predictor of IA, *β* = −.56, *t* = −3.12, *p* = .004. When autistic trait scores were entered in step 2 and alexithymia in step 3, autistic trait scores significantly improved the model, *F*(1,27) = 6.03, *p* = .021, accounting for an additional 13.6% of the variance in IA. Importantly, however, in step 3, alexithymia was a significant predictor of IA, *β* = −.56, *t* = −3.12, *p* = .004, while autistic trait scores were no longer predictive (*p* = .37). Furthermore, the addition of alexithymia led to a significant change in *R*^2^, increasing the variance in IA accounted for by 16.6%, *F*(1,26) = 9.76, *p* = .004. These results are consistent with and extend those of Experiment 1: Groupwise comparisons and regression analyses converged to indicate that alexithymia, and not autism, is associated with impaired interoception.

## 4 General discussion

The present study examined the relative impact of autism and alexithymia on interoception. A well-established measure of IA, together with a TE control procedure was used, and a number of clinically relevant variables previously reported to be related to interoception were recorded. In a non-clinical sample, autistic traits were unrelated to IA. In contrast, alexithymia was – even after accounting for confounding variables – a significant predictor of IA (Experiment 1). It was then found that, after controlling for alexithymia, there is no evidence for impaired interoception in ASD (Experiment 2). Furthermore, alexithymia was the strongest predictor of IA, above and beyond demographic variables and other factors potentially related to interoception.

These results represent an important contribution to the growing literature on interoceptive difficulties in autism, alexithymia and other clinical conditions. The correlation between alexithymia and interoception in the non-clinical sample (*r* = −.36; Experiment 1) matched that from a larger non-clinical study (*r* = −.37, *n* = 155; Herbert et al., 2011). Collapsing across the ASD and control groups, this relationship was even larger in Experiment 2, presumably because of increased variance in alexithymia. This is consistent with findings that neural systems supporting interoception (e.g., Critchley, Wiens, Rotshtein, Öhman, & Dolan, 2004) may be atypical in people with alexithymia (Ernst et al., 2014). The robustness of the relationship between alexithymia and interoception, and the lack of an association between interoception and ASD, suggests that previous findings of reduced interoceptive ability in ASD are, in fact, a consequence of the greater proportion of alexithymic individuals in the ASD population. It supports Brewer et al.'s (2015) suggestion that, where observed in ASD, signs of atypical interoception are due to co-occurring alexithymia. A higher incidence of alexithymia in numerous psychiatric and neurological disorders (Bird and Cook, 2013, Ricciardi et al., 2015) supports the inclusion of alexithymia measures when conducting interoception research in any clinical group.

Clinically, these results have implications for diagnosis (in establishing which symptoms of clinical conditions are associated with the clinical condition *per se*, and which may be caused by co-occurring alexithymia), and for intervention. They suggest interoceptive training may be therapeutic (e.g., Schaefer, Egloff, Gerlach, & Witthöft, 2014), but potentially only for those patients with co-occurring alexithymia. Given the hypothesized benefit of oxytocin for interoceptive conditions (Quattrocki & Friston, 2014), it is notable that beneficial effects of oxytocin may be limited to those with high levels of alexithymia (Luminet, Grynberg, Ruzette, & Mikolajczak, 2011). However, it may also be the case that alexithymic individuals have such diminished interoception that interoceptive training programs prove to be unsuccessful. These possibilities are worthy of further investigation as they will help to elucidate the circumstances in which interoceptive training is therapeutically beneficial.

Two caveats are worthy of consideration with respect to these results. First, there remain difficulties with the measurement of interoception. Although the Heartbeat Tracking task is widely used, in some individuals heartbeat may be perceived via discriminative (exteroceptive) touch receptors due to the vibration of the chest wall (Khalsa, Rudrauf, Feinstein, & Tranel, 2009). Future research into (atypical) interoception should therefore employ multiple measures in order to provide convergent information on interoceptive ability (Garfinkel et al., 2015). Second, this study was designed specifically to elucidate the relative impact of autism and alexithymia on interoceptive ability, while statistically controlling for other clinical traits thought to be related to interoception (e.g., anxiety). Nevertheless, in accordance with existing research, we observed trend-level indications of a relationship between anxiety and IA (see Paulus & Stein, 2010). In future research a sample comprising sufficient variance on these traits will enable assessment of the independent impact of, and interrelationships between, these variables and IA.

In summary, across two experiments, results suggest that alexithymia, not autism, is associated with atypical IA, even after controlling for several clinical traits associated with interoception. This advances recent research into interoceptive dysfunction in ASD, and suggests that the interoceptive account of ASD may require revision. However, interpreting atypical behavior within an interoceptive framework has potential to inform the clinical management of autism and other psychiatric conditions.

## Footnotes

1 The use of the term ‘autistic’ is endorsed by many individuals with ASD (see Kenny et al., 2015). We therefore use this term as well as language preferred by clinical professionals (e.g., ‘individuals with autism’) to respect the wishes of autistic individuals and report the study in line with scientific parlance.
