# Supplementary material for: Alexithymia, not autism, is associated with impaired interoception
Source: Cortex. 2016 Aug;81:215–20. doi: 10.1016/j.cortex.2016.03.021 (PMC4962768; doi:10.1016/j.cortex.2016.03.021)
Supplement: Supplementary file 1 [file mmc1.doc]

**Supplemental Information:
Alexithymia, not autism, is associated with impaired interoception**

Punit Shah, Richard Hall, Caroline Catmur, & Geoffrey Bird

**Experiment 1**

Table S1

Correlations between Interoceptive Accuracy and other Variables

|  | IA | TAS-20 | AQ | BDI | TA | SA | BMI | TE |
| --- | --- | --- | --- | --- | --- | --- | --- | --- |
| IA |  | – 0.36^*^ | – 0.09 | 0.06 | 0.15 | – 0.10 | – 0.10 | – 0.001 |
| TAS-20 |  |  | 0.56^***^ | 0.22 | 0.09 | 0.23 | – 0.04 | – 0.14 |
| AQ |  |  |  | 0.55^***^ | 0.45^**^ | 0.40^*^ | 0.19 | – 0.02 |
| BDI-II |  |  |  |  | 0.59^***^ | 0.35^*^ | 0.32^*^ | 0.09 |
| TA |  |  |  |  |  | 0.59^***^ | 0.15 | 0.09 |
| SA |  |  |  |  |  |  | 0.08 | 0.10 |
| BMI |  |  |  |  |  |  |  | 0.05 |

*Note*. ^*^ *p* < .05, ^**^ *p* < .01, ^***^ *p* < .001. Interoceptive Accuracy (IA), the 20-item Toronto Alexithymia Scale (TAS-20; Bagby, Parker, & Taylor, 1994), Autism-spectrum Quotient (AQ; Baron-Cohen et al., 2001), Beck Depression Inventory (BDI-II; Beck, Steer, & Brown, 1996), the Spielberger State Anxiety (SA)/Trait Anxiety (TA) Inventory (Spielberger, Gorsuch, Lushene, Vagg, & Jacobs, 1983), Body Mass Index (BMI) and Time Estimation score (TE).

**Experiment 2**

Thirty-eight right-handed adults with (*n* = 19) and without autism (*n* = 19) participated in Experiment 2. Participants with Autism Spectrum Disorder (ASD) were recruited from a database held at the Institute of Cognitive Neuroscience, University College London, United Kingdom. All had received independent clinical diagnosis (according to the DSM-IV; American Psychiatric Association, 1994) of an ASD from an independent clinical practitioner. All autistic participants also met the criteria for autism or ASD on the Autism Diagnostic Observational Schedule (ADOS; Lord et al., 2000). All participants completed the Autism-spectrum Quotient AQ (Baron-Cohen et al., 2001) to measure autistic traits, on which the ASD group scored higher than the matched control group (Table S2). Finally, all participants reported normal or corrected-to-normal vision.

Table S2

Mean Age, Gender, Autism-spectrum Quotient (AQ), 20-Item Toronto Alexithymia Scale (TAS-20) and IQ scores (Wechsler, 1997) for the Autism Spectrum Disorder (ASD) and matched neurotypical control group. Autism Diagnostic Observational Schedule (ADOS) score and classification details for the ASD group.

|  | **ASD** | **Controls** | **Comparison** |
| --- | --- | --- | --- |
| N | 19 | 19 | - |
| Gender | 15 Male, 4 Female | 13 Male, 6 Female | χ^2^(1) = 0.54,  *p* = .46 |
| Mean Age (Years) | 32.89 (11.49) | 32.89 (14.41) | *p* > .99, *d* < 0.01 |
| Mean Full-scale IQ | 109.00 (12.99) | 111.89 (13.17) | *p* = .50, *d* = 0.22 |
| Mean AQ | 35.26 (7.40) | 19.32 (8.37) | *p* < .001, *d* = 2.02 |
| Mean TAS-20 | 58.11 (13.00) | 55.37 (18.33) | *p* = .60, *d* = 0.17 |
| Number of participants with / without alexithymia | 7 / 12 | 10 / 9 | χ^2^(1) = 0.96,  *p* = .33 |
| ADOS Classification | 7 Autism, 12 Autism Spectrum | - | - |
| Mean ADOS Score | 9.16 (2.12) | - | - |

*Note*. ADOS score is derived from an algorithm (Lord et al., 2000) with a higher score representing a higher degree of autism. Standard deviations are shown in parentheses.

Table S3

Correlations between Interoceptive Accuracy and other Variables (Collapsing Across Groups)

|  | IA | TAS-20 | AQ | BDI | TA | SA | BMI | TE |
| --- | --- | --- | --- | --- | --- | --- | --- | --- |
| IA |  | – 0.64^***^ | – 0.20 | –0.16 | 0.19 | – 0.05 | 0.25 | – 0.05 |
| TAS-20 |  |  | 0.37^*^ | 0.23 | 0.02 | 0.17 | – 0.26 | – 0.29 |
| AQ |  |  |  | 0.42^*^ | 0.52^**^ | 0.38^*^ | 0.26 | 0.07 |
| BDI-II |  |  |  |  | 0.54^**^ | 0.55^***^ | 0.21 | 0.19 |
| TA |  |  |  |  |  | 0.70^***^ | 0.26 | 0.06 |
| SA |  |  |  |  |  |  | 0.13 | 0.03 |
| BMI |  |  |  |  |  |  |  | 0.10 |

*Note*. ^*^ *p* < .05, ^**^ *p* < .01, ^***^ *p* < .001. Interoceptive Accuracy (IA), the 20-item Toronto Alexithymia Scale (TAS-20; Bagby et al., 1994), Autism-spectrum Quotient (AQ; Baron-Cohen et al., 2001), Beck Depression Inventory (BDI-II; Beck et al., 1996), the Spielberger State Anxiety (SA)/Trait Anxiety (TA) Inventory (Spielberger et al., 1983), Body Mass Index (BMI) and Time Estimation score (TE).

**Supplemental References**

American Psychiatric Association. (1994). *Diagnostic and Statistical Manual of Mental Disorders (DSM-IV)*. Washington, DC: Author.

Bagby, R. M., Parker, J. D., & Taylor, G. J. (1994). The twenty-item Toronto Alexithymia Scale--I. Item selection and cross-validation of the factor structure. *Journal of Psychosomatic Research*, *38*(1), 23–32.

Baron-Cohen, S., Wheelwright, S., Skinner, R., Martin, J., & Clubley, E. (2001). The autism-spectrum quotient (AQ): evidence from Asperger syndrome/high-functioning autism, males and females, scientists and mathematicians. *Journal of Autism and Developmental Disorders*, *31*(1), 5–17.

Beck, A. T., Steer, R. A., & Brown, G. K. (1996). *Beck Depression Inventory-II*. San Antonio, TX: Psychological Corporation.

Lord, C., Risi, S., Lambrecht, L., Cook, E. H., Leventhal, B. L., DiLavore, P. C., … Rutter, M. (2000). The autism diagnostic observation schedule-generic: a standard measure of social and communication deficits associated with the spectrum of autism. *Journal of Autism and Developmental Disorders*, *30*(3), 205–223.

Spielberger, C. D., Gorsuch, R. L., Lushene, R., Vagg, P. R., & Jacobs, G. A. (1983). *Manual for the State-Trait Anxiety Inventory*. Palo Alto, CA: Consulting Psychologists Press.

Wechsler, D. (1997). *Wechsler Adult Intelligence Scale (3rd Edition)*. San Antonio, TX: Psychological Corporation.
